# EnViTSA: Ensemble of Vision Transformer with SpecAugment for Acoustic Event Classification

**DOI:** 10.3390/s23229084

**Published:** 2023-11-10

**Authors:** Kian Ming Lim, Chin Poo Lee, Zhi Yang Lee, Ali Alqahtani

**Affiliations:** 1 Faculty of Information Science and Technology, Multimedia University, Melaka 75450, Malaysia; kmlim@mmu.edu.my; 2 DZH International Sdn. Bhd., Kuala Lumpur 55100, Malaysia; 3 Department of Computer Science, King Khalid University, Abha 61421, Saudi Arabia; amosfer@kku.edu.sa; 4 Center for Artificial Intelligence (CAI), King Khalid University, Abha 61421, Saudi Arabia

**Keywords:** acoustic event classification, log mel-spectrograms, specaugment, ensemble learning, vision transformer

## Abstract

Recent successes in deep learning have inspired researchers to apply deep neural networks to Acoustic Event Classification (AEC). While deep learning methods can train effective AEC models, they are susceptible to overfitting due to the models’ high complexity. In this paper, we introduce EnViTSA, an innovative approach that tackles key challenges in AEC. EnViTSA combines an ensemble of Vision Transformers with SpecAugment, a novel data augmentation technique, to significantly enhance AEC performance. Raw acoustic signals are transformed into Log Mel-spectrograms using Short-Time Fourier Transform, resulting in a fixed-size spectrogram representation. To address data scarcity and overfitting issues, we employ SpecAugment to generate additional training samples through time masking and frequency masking. The core of EnViTSA resides in its ensemble of pre-trained Vision Transformers, harnessing the unique strengths of the Vision Transformer architecture. This ensemble approach not only reduces inductive biases but also effectively mitigates overfitting. In this study, we evaluate the EnViTSA method on three benchmark datasets: ESC-10, ESC-50, and UrbanSound8K. The experimental results underscore the efficacy of our approach, achieving impressive accuracy scores of 93.50%, 85.85%, and 83.20% on ESC-10, ESC-50, and UrbanSound8K, respectively. EnViTSA represents a substantial advancement in AEC, demonstrating the potential of Vision Transformers and SpecAugment in the acoustic domain.

## 1. Introduction

Sound is a mechanical wave produced by the vibrations of air molecules through a transmission medium, such as air. Various sound sources generate acoustic waves with distinct attributes, including pitch, duration, loudness, timbre, sonic texture, and spatial location [1]. Acoustic Event Classification (AEC) aims to categorize acoustic events into specific classes, a task that humans perform effortlessly but machines find challenging. Acoustic event classification has been widely applied in audio surveillance [2], ambient assisted living [3], content-based multimedia retrieval [4], and bio-acoustics monitoring [5]. With the advancements in deep learning, recent works in AEC have achieved state-of-the-art performance using deep neural networks. However, AEC remains challenging due to background noise, variations in the length of acoustic signals, data scarcity, and other factors. An acoustic signal is a sequence of samples ordered in the time domain, making it a form of temporal data.

Generally, there are two main approaches to acoustic event classification: hand-crafted approaches [6,7,8,9] and deep learning-based approaches [10,11,12]. The hand-crafted approach involves extracting features from acoustic signals and using a classifier to categorize these features into the correct classes. During feature extraction, a set of robust features is selected from the acoustic signal. These selected features form a feature vector enriched with meaningful information about the acoustic signal, significantly reducing the dimensionality of the acoustic data. Subsequently, these extracted features are fed into a classifier for classification. Therefore, the quality of the extracted features and the classifier greatly impact the performance of the AEC model. Conversely, the deep learning-based approach learns an AEC model in an end-to-end manner. Deep learning methods have the capability to automatically learn significant representations from acoustic signals, eliminating the need for manually engineered features that may not be robust to unseen data. In light of this, we propose a deep learning-based acoustic event classification model, referred to as the Ensemble of Vision Transformer with SpecAugment (EnViTSA).

In general, raw acoustic samples vary in length. However, deep learning-based approaches require a fixed input. From the signal domain perspective, audio signals can be represented in the time domain, frequency domain, and time-frequency domain [13]. In this work, we employ time-frequency representation via spectrogram to obtain a robust representation of the acoustic signal with a fixed length. Over the past decade, Convolutional Neural Network (CNN)-based models [10,11] have found widespread use in Acoustic Event Classification (AEC). However, CNN-based networks inherently rely on two inductive biases: the assumption that neighboring pixels are related (locality) and that different parts of input data should be processed in the same way regardless of their absolute position (weight sharing). Furthermore, CNN-based networks demand a large number of training samples to build effective models. Yet, real-world applications consistently face data scarcity issues. With limited training samples, CNN-based networks are susceptible to overfitting. To address these challenges, we introduce the Ensemble of Vision Transformers as an approach to minimize inductive biases and effectively reduce overfitting. Additionally, we employ SpecAugment as a means to generate more training samples, mitigating the data scarcity problem and substantially enhancing the AEC model’s performance. The main contributions of this paper include:

- Conversion of Variable-Length Acoustic Samples: mitigating the varying length of the acoustic samples by converting it into a fixed length time-frequency spectrogram representation.
- Ensemble of Vision Transformers: proposing an ensemble of pre-trained Vision Transformers, a novel approach that minimizes inductive biases and effectively reduces overfitting.

This paper is organized as follows: Section 2 discusses related work in acoustic event classification. Section 3 provides a detailed description of the proposed Ensemble of Vision Transformer with SpecAugment. Section 4 presents the experimental results and analysis. Lastly, we draw some conclusions in Section 5.

## 2. Related Work

In general, existing research in acoustic event classification can be categorized based on signal representations, data augmentation techniques, network architectures, and learning paradigms.

In order to improve the performance of AEC, several works have been explored on robust signal representations. In [14], the effectiveness of various time-frequency domain representations of audio was examined, including linear-scaled Short-Time Fourier Transform (STFT) spectrogram, Mel-scaled STFT spectrogram, constant-Q transform spectrogram, and continuous wavelet transform spectrogram. In their work, they found out that all time-frequency representations generally outperform Mel-frequency cepstral coefficients (MFCC) features. According to the author [14], the wideband Mel-scaled STFT spectrogram exhibited subpar performance when dealing with short-duration sounds such as “drilling” or “jackhammer,” as well as continuous, monotonous sounds such as “air conditioner”. However, it demonstrated superior results when handling classes characterized by significant variations in high-frequency components, such as “children playing”. In contrast, the narrowband Mel-scaled STFT spectrogram offered enhanced temporal resolution but diminished frequency accuracy, exhibiting the opposite behavior. Later, a convolutional restricted Boltzmann machine (ConvRBM) [15] was used for unsupervised filter bank learning from raw audio signals. The learned feature (ConvRBM-BANK) outperformed other baseline features such as Mel filterbank energies and gammatone spectral coefficients. A recent work [16] combined handcrafted features and deep features that were extracted from a CNN. These works have shown that time-frequency domain representation as in a spectrogram can produce a fixed-length robust representation for AEC.

To address the issue of limited training samples in AEC, some studies have applied data augmentation techniques to augment the available training data. In [17], the authors shown the importance of preserving the original meaning of the class labels after data augmentation had been performed, and suggested the use of separate data augmentation techniques for each class. Later, ref. [18] introduced the concept of between-class learning, drawing inspiration from mix-up techniques commonly used in image classification. In their approach, they did not generate additional training samples in the conventional sense. Instead, each training sample was created by mixing two random training samples from the original datasets. This strategy aimed to enhance Fisher’s criterion and introduce a regularization effect on the locational correlation of feature distributions. A similar approach was presented in [19] with a simpler mixing method. More recently, a study by Mushtaq et al. [20] compared the performance of two different approaches: conventional image augmentation applied directly to spectrograms and non-image augmentation applied to the audio clips, which were subsequently converted into spectrograms. The results demonstrated that the latter approach generally yielded better results than the former. Collectively, these works highlight the utility of data augmentation in generating synthetic training samples to enhance the performance of AEC models.

Inspired by the successes of deep neural networks applied in various tasks [21], researchers have explored different network architectures to assess their effectiveness in AEC. In [22], traditional small convolution filters in CNNs were replaced with dilated filters to expand the receptive field, enabling the capture of more contextual information. They also utilized LeakyReLU to preserve information from negative inputs that might be valuable. In order to address the problem of fluid temporal properties of environmental sounds, [23] incorporated temporal attention into the three-stream network architecture. The network received three inputs, which are 1D raw waveforms, 3D stacked STFT spectrograms, and 3D delta features from STFT spectrograms. Temporal attention is capable of reducing the background noise and finding the temporal patterns within the acoustic signals. Later, temporal attention and channel attention techniques were proposed in [24]. Their model effectively captured complex temporal structures and diverse energy modulation patterns of environmental sounds. They employed a convolutional recurrent neural network that received 3D features generated by combining static Log gammatone-spectrograms with delta information. In the work by [25], distinct audio features were used in each channel of the four-channel inputs fed into a handcrafted CNN model. These features included constant Q-transform, chromagram, Gammatone Frequency Cepstral Coefficients (GFCC), and MFCC. Additionally, they incorporated an attention mechanism and mix-up techniques into their model. While most of these works focus on deep neural network-based architectures, it is important to note that the performance of these networks is significantly influenced by inherent inductive biases.

Several studies have explored the combination of conventional handcrafted approaches and deep learning techniques to enhance model performance in AEC. In [26], a handcrafted CNN was proposed to extract deep features from spectrograms, which were subsequently input into an ensemble of K-nearest neighbors (KNN) models for classification. A similar approach [27] employed three pre-trained models, namely VGGNet16, VGGNet19, and DenseNet201, for feature extraction from spectrograms in a pyramidal fashion. These features were then aggregated and classified using a Support Vector Machine (SVM) model. The increasing popularity of transfer learning in recent years has motivated some researchers to adopt pre-trained models from the image domain for AEC tasks. In [28], ESResNet was proposed as an extension of ResNet, featuring an additional attention block. The authors demonstrated that fine-tuning a pre-trained network yielded better results than training the same network from scratch. Subsequently, the feasibility of using models pre-trained on image datasets in the audio domain was investigated in [29]. The authors showed that pre-trained weights, even from inputs of a different domain, were valuable, particularly in the initial layers. Combination of handcrafted approaches and deep learning approaches shows improvement in the performance of AEC. However, this strategy requires thoroughly fine-tuning on the adopted pre-trained models.

## 3. Ensemble of Vision Transformer with SpecAugment

**Figure 1:**
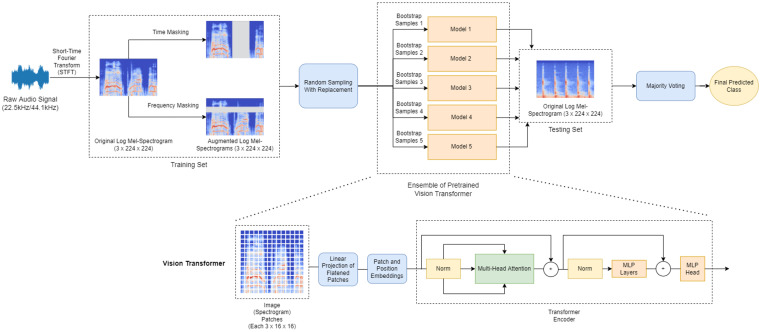
The proposed Ensemble of Vision Transformer with SpecAugment for acoustic event classification.

In this work, an ensemble of Vision Transformers with SpecAugment for acoustic event classification, named EnViTSA, is proposed. The proposed EnViTSA is depicted in Figure 1. Given the input raw audio signal, it is first converted into a time-frequency representation, i.e., Log Mel-spectrograms. In order to increase the training samples and mitigate the data scarcity problem, SpecAugment is proposed to perform data augmentation on the obtained Log Mel-spectrograms. The resulting training sets with augmented samples are then fed into the proposed ensemble of Vision Transformers for training. Five Vision Transformers are employed to train on the sub-samples produced from random sampling with replacement. In doing so, the issue of overfitting when only a single model was used can be addressed. Majority voting is utilized to classify the sample into the correct class.

### 3.1. Time-Frequency Representation

Audio signals can be represented in three different domains: time domain, frequency domain, and time-frequency domain. In this work, the time-frequency representation of audio, the spectrogram, has been used. The spectrogram is generated by applying Short-Time Fourier Transform (STFT) to the raw audio waveform.

STFT can be thought of as applying the Discrete Fourier Transform (DFT) to each frame of the signal one at a time. The result of the STFT is a collection of complex Fourier coefficients for each of the frames. A window function is applied to the original signal to generate individual windows from the signal. The window operation can be described by the formula:

(1) $$x_{w}\left(n\right)=x\left(n\right)\times w\left(n\right)$$

where $x_{w}\left(n\right)$ is the windowed signal, x(n) is the original signal, w(n) is the window function, and each of them is represented as a function of sample *n*. The most common type of window function is the Hann window, which is also used in this work. The formula of the Hann window is:

(2) $$w\left(n\right)=0.5\times \left(1-\cos\left(\frac{2\pi n}{N-1}\right)\right),\;\mathrm{for}\;0\leq n\leq N-1$$

where *N* is the total number of samples in the signal.

Some important parameters that affect the STFT include frame size, window size, and hop length. The window size refers to the number of samples that the windowing operation is applied to. Windows in an audio signal can overlap with each other. Hence, the hop length represents the number of samples that slide to the right from one window to the next window. Frame size refers to the number of samples inside a frame on which STFT is to be applied. Under normal circumstances, frame size and window size are set to a common value, although sometimes frame size can be larger than window size. The formula for STFT is given as:

(3) $$S\left(m,k\right)=\sum_{n=-\infty }^{\infty }x\left(n+mH\right)w\left(n\right)e^{-i2\pi nk/N}$$

where S(m,k) is the complex STFT coefficient for the *m*th frame at the *k*th frequency, *N* is the number of samples inside a frame (frame size), *H* is the hop size, and w(n) is the window function.

The squared magnitude (absolute value) of the complex coefficient is taken to extract useful information from the STFT result. It provides information about how much power a particular frequency has contributed to the signal at a particular frame. In contrast, the magnitude of the complex coefficient provides information about the energy.

Since the frames overlap, the output of STFT consists of a different number of frames for signals of varying lengths. To ensure a fixed-length representation, the spectrogram frames are concatenated into a longer matrix or shorter signals are padded with zeros to match the desired length. When STFT is applied across all the frames and frequencies, the result is a 2D matrix that can be plotted as a heat map, which is the spectrogram. The horizontal axis of the spectrogram represents the frames, which approximates continuous time. The vertical axis represents the frequency bins, which approximates continuous frequency. Each point in the spectrogram shows the power (amplitude squared) of a particular frequency bin at a specific frame, indicated by different colors. The higher the power, the brighter the color.

However, the spectrogram generated in this manner often lacks the visual clarity needed for human interpretation. This is because human perception of sound intensity, which is proportional to amplitude squared, as well as frequency, follows a logarithmic scale rather than a linear one. To address this, the power is converted into decibel (dB) units, and the linear frequency axis is transformed into a logarithmic scale. The outcome of this transformation is referred to as the Log spectrogram [30].

Finally, the Log Mel-spectrogram is the spectrogram that uses dB units for the power (amplitude), and the frequency axis uses the Mel scale. The Mel scale accounts for the way humans perceive frequency in a logarithmic manner. For example, humans perceive the distance from 100 Hz to 200 Hz as greater when compared to the distance from 1000 Hz to 1100 Hz, even though the difference in frequencies is the same, which is 100 Hz. Hertz can be converted into Mels using the following formula:

(4) $$m=2595\log_{10}\left(1+\frac{f}{700}\right)$$

where *m* is the Mel value, and f is the frequency measured in Hz.

### 3.2. SpecAugment

Data augmentation is an essential technique used to increase the size of datasets, which can prevent overfitting and improve the performance of deep learning models. The three datasets used in this work have a limited number of samples, which makes them prone to overfitting. To overcome this limitation, a set of data augmentation techniques called SpecAugment, specifically designed for spectrograms, is proposed. SpecAugment is introduced in [31] and consists of three variations: time warping, time masking, and frequency masking.

Time warping introduces horizontal deformations to the spectrogram frames, shifting them either to the right or left. Time masking entails the insertion of silent blocks spanning specific time intervals, while frequency masking involves the insertion of silent blocks over designated frequency channels. In our experimentation, it became evident that time warping made a negligible contribution to overall performance improvement. Consequently, we opted to solely employ time masking and frequency masking as augmentation techniques, applying them uniformly to every training sample in the dataset. This augmentation process significantly bolsters the dataset’s diversity and generalization capabilities, ultimately enhancing the model’s robustness against overfitting.

**Figure 2:**
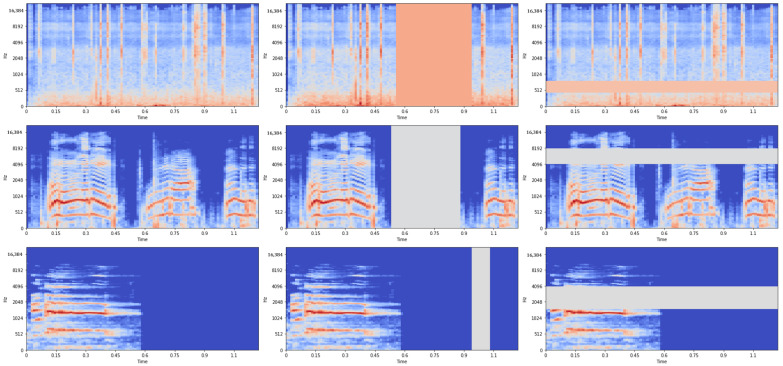
Spectrogram examples of ESC-10 with no augmentation (**left**), time masking (**middle**), and frequency masking (**right**).

To illustrate the effect of data augmentation, Figure 2 displays nine random examples from the ESC-10 dataset: one with no augmentation (left), one after time masking (middle), and one after frequency masking (right). The figure demonstrates that the inclusion of time and frequency masking introduces variations in the spectrogram. These variations can assist the model in learning more robust features and enhancing its performance.

### 3.3. Vision Transformer

The Transformer architecture, initially introduced by Vaswani et al. in 2017 [32] to tackle machine translation tasks, has since gained widespread acceptance in the field of natural language processing (NLP). One of the primary advantages of the Transformer is its elimination of the need for recurrent operations, which were essential in previously popular NLP models, e.g., Recurrent Neural Networks (RNNs) and Long Short-Term Memory (LSTM) networks.

At the core of the Transformer architecture is the self-attention mechanism. Self-attention can be likened to the process of concentrating on specific elements within a sequence that hold vital roles in comprehending that sequence. It is an intuitive mechanism akin to how humans perform tasks such as understanding, translating, and summarizing a paragraph. Self-attention can also be likened to matching a query with a set of keys, where each key corresponds to its unique values.

Despite its popularity in NLP, the Transformer architecture was not widely adopted in the field of computer vision. Instead, researchers attempted to introduce self-attention mechanisms into existing Convolutional Neural Network (CNN) architectures. However, self-attention could not be implemented directly for images due to the large number of pixels in an image. To address this problem, Dosovitskiy et al. proposed the Vision Transformer (ViT) architecture [33], which can be used directly for general computer vision tasks. The architecture of the ViT is shown in Figure 1.

One of the main features of the ViT is that it breaks down an image into a sequence of constant-sized patches, each of which is embedded with its corresponding position embedding to denote its position. The ViT also includes the addition of a learnable class embedding at the front of the sequence, which is then fed into the Transformer encoder. The output of the MLP head is the probabilities with which an image belongs to each of the classes.

Since the ViT learns a large number of parameters, it is often adopted using transfer learning to avoid overfitting. In this work, a pretrained ViT on ImageNet is employed. Each input image is divided into patches of size 3×16×16 from the original size of 3×224×224. The number of nodes in the MLP head is adjusted according to the number of classes in the dataset, which is 10 for UrbanSound8K/ESC-10 and 50 for ESC-50.

The use of the ViT for acoustic event classification is justified by the fact that sound events usually span across time and are encoded in spectrograms, which are two-dimensional representations of sound signals. The horizontal axis of the spectrogram represents time, while the vertical axis represents frequency. Thus, it is crucial for a model to capture the temporal information in spectrograms. While CNNs focus on local information, the self-attention mechanism in the ViT enables the model to derive global information from inputs, making it a better fit for capturing temporal information in spectrograms [34].

### 3.4. Ensemble Learning

Ensemble learning is a technique that has gained significant popularity in recent years due to its ability to achieve state-of-the-art results across various problem domains [35]. The basic idea of ensemble learning is to combine the outputs of several individual models, or base models, in order to improve the overall decision-making process. This approach assumes that the collective decision of multiple models is more robust and accurate than a decision made by a single model. Ensemble learning methods can be categorized based on the way they combine the predictions of individual models. Bagging, boosting, random forest, and stacking are some examples of ensemble learning methods. In this work, the bagging technique has been employed to address the issue of overfitting, which is a common problem in machine learning when only a single model is used.

The bagging technique involves training multiple base models independently on different subsets of the training data. These subsets are generated through random sampling with replacement, meaning that a sample can be selected more than once in the same subset. This process of sampling is known as bootstrap sampling. The goal is to create a diverse set of base models that can learn different aspects of the data. After training, the individual models make predictions on the test set, and their outputs are combined using a majority voting scheme. The class that is predicted most frequently by the individual models is assigned as the final predicted class.

In this work, five Vision Transformer models have been trained using the bagging technique, each on a different subset of the training data. The number of members in the ensemble model is varied from 1 to 5 to investigate its impact on the classification performance. The results show that as the number of members in the ensemble model increases, the classification performance generally improves. However, this is not always the case, as the effectiveness of ensemble learning relies on both the diversity and predictive power of the member models. After every Vision Transformer model has been trained on its distinctive bootstrap samples, the classification results are combined using majority voting, in which classes that are most frequently predicted by the member models are assigned as the final predicted classes. By combining the outputs of several base models, ensemble learning can overcome the limitations of individual models, prevent overfitting, and produce more accurate predictions.

## 4. Experiment Results and Analysis

In this section, the three benchmark datasets that used in this work and the experiment results and analysis are presented.

### 4.1. Datasets

Three datasets used in this work are UrbanSound8K [36], ESC-50, and ESC-10 [37].

#### 4.1.1. UrbanSound8K

**Figure 3:**
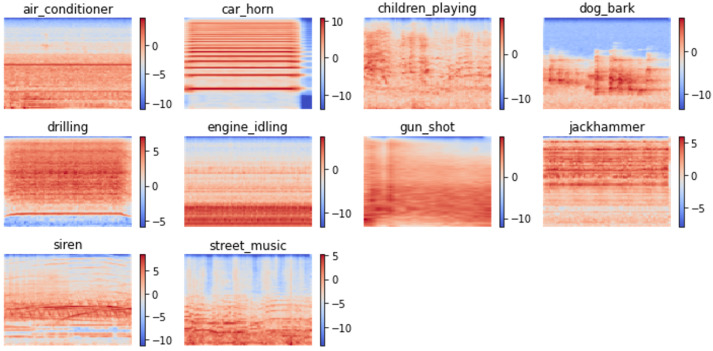
Sample of generated spectrograms from the UrbanSound8K dataset.

UrbanSound8K is a collection of 8732 audio slices that record sounds occurring in an urban environment. It contains a total of 10 classes, including air conditioner, car horn, children playing, dog bark, drilling, engine idling, gun shot, jackhammer, siren, and street music, each of which corresponds to class labels 0 to 9 used in this work. The class distribution is imbalanced, with each of the first seven classes having 1000 samples, while the class jackhammer has 929 samples, the class siren has 429 samples, and the class street music has 374 samples. Every audio slice has varying durations of not more than 4 s. The total duration of all audio slices is 8.75 h. The dataset is available in the form of 10 predefined folds. Audio slices originating from the same recording are placed under the same fold, and each fold is ensured to have a similar class distribution. Figure 3 shows randomly generated spectrogram samples of each class in UrbanSound8K.

#### 4.1.2. ESC-50

**Figure 4:**
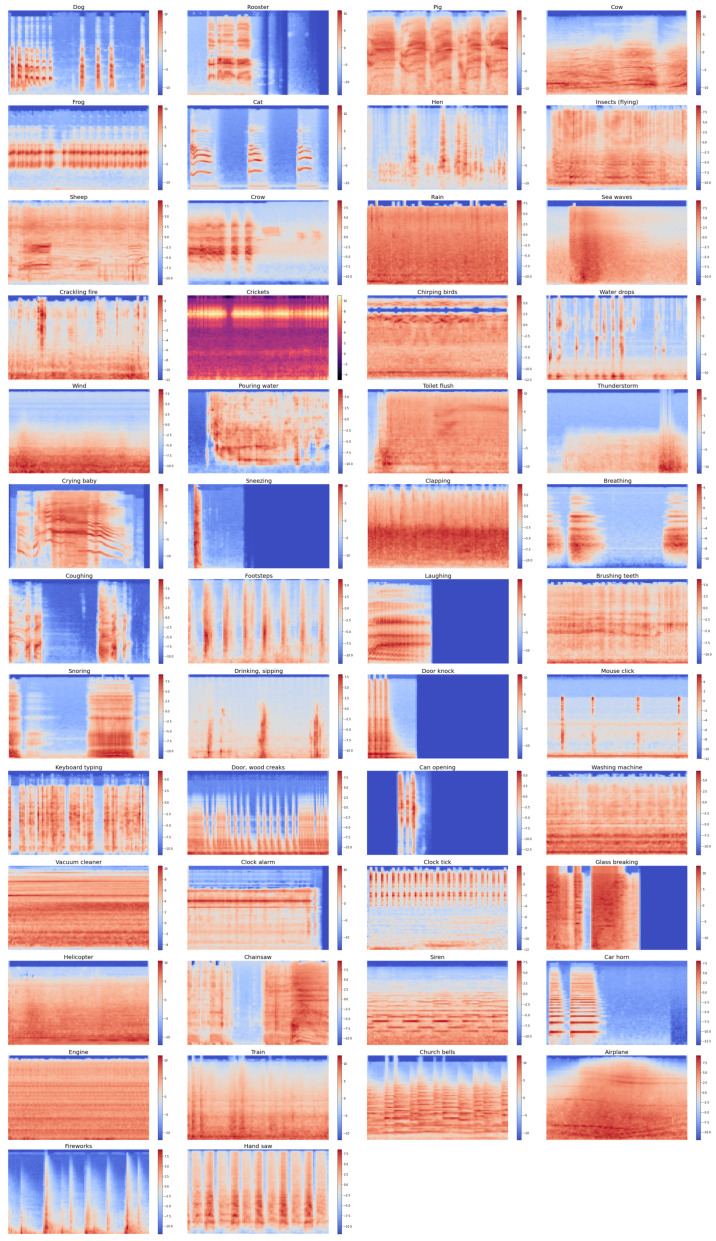
Sample of generated spectrograms from the ESC-50 dataset.

ESC-50 is a collection of 2000 audio clips containing environmental sounds from 50 different classes. The dataset is balanced, with each class having 40 samples. Each audio clip is converted into a single-channel, 44,100 Hz format using Ogg Vorbis compression at a rate of 192 kbit/s and has a duration of 5 s. The dataset is provided in the form of five predefined folds, with audio clips from the same recording being placed in the same fold. Figure 4 displays randomly selected spectrogram samples from each class in the ESC-50 dataset.

#### 4.1.3. ESC-10

**Figure 5:**
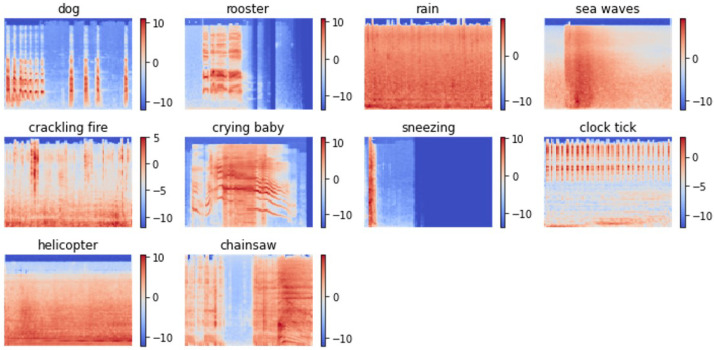
Sample of generated spectrograms from the ESC-10 dataset.

ESC-10 is a subset of ESC-50, containing 400 samples with 40 samples in each of the 10 classes. These classes are dog, rooster, rain, sea waves, crackling fire, crying baby, sneezing, clock tick, helicopter, and chainsaw, corresponding to class labels 0 to 9 in this work. These classes can generally be classified into three groups: transient/percussive sounds, sound events with strong harmonic content, and structured noise or soundscapes. Figure 5 displays randomly selected spectrogram samples from each class in ESC-10.

### 4.2. Training of EnViTSA and the Performance Evaluation Metric

**Figure 6:**
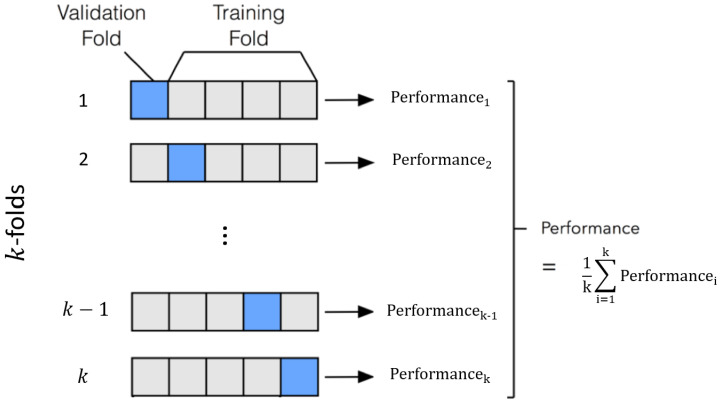
*k*-fold cross validation.

Algorithm 1 presents the training procedure for the proposed EnViTSA in acoustic event classification. In this work, we utilize *k*-fold cross-validation to assess performance. As the name implies, *k*-fold cross-validation is a variant of traditional cross-validation. The dataset is divided into *k* equally sized folds. The training and validation process is repeated *k* times, where in each iteration, one of the *k* folds is designated as the validation set, while the remaining k−1 folds serve as the training set. In essence, each fold serves as the validation set once and as part of the training set k−1 times. The final result is an average of the results obtained across all *k* iterations, as illustrated in Figure 6. The choice of *k* is arbitrary but commonly set to 5 or 10. The specific value of *k* often corresponds to the naming convention, such as 5-fold cross-validation for k=5. It is worth noting that, in the context of *k*-fold cross-validation, the terms “validation” and “testing” are frequently used interchangeably. For this study, we adhere to the established practice found in previous works [17,18,28,38,39,40,41,42,43,44,45], where 10-fold cross-validation is applied to UrbanSound8K, and 5-fold cross-validation is applied to ESC-10 and ESC-50.

In addition to *k*-fold cross-validation, the logic necessary for ensemble learning must be integrated. Specifically, within each *k*-th iteration, the model is trained independently five times, resulting in five distinct member models for inclusion in the final ensemble model. The prediction results generated by each member model are subsequently aggregated incrementally, as explained in the previous section. This process allows us to calculate the validation accuracy for the five Vision Transformer models in each *k*. Following *k* iterations of training, the validation accuracies obtained for each model are averaged by *k*, resulting in five validation accuracy values. Each of these values corresponds to the *k*-fold cross-validation result when each model is utilized. The final result is determined by selecting the maximum value from these validation accuracies.
**Algorithm 1** EnViTSA: Ensemble of Vision Transformer with SpecAugment for acoustic event classification 1:**Input**:
Training dataset $D_{train}$ with audio samples and labelsNumber of ensemble members *N*Number of epochs *E*Number of folds *K* 2:**Output**:
Trained ensemble of *N* Vision Transformer models 3:Initialize an empty ensemble list ensemble←[] 4:**for** *i* from 1 to *N* **do** 5:   **for** *k* from 1 to *K* **do** 6:     Split $D_{train}$ into training set $D_{train_{k}}$ and validation set $D_{val_{k}}$ using fold *k* 7:     Preprocess $D_{train_{k}}$ using SpecAugment 8:     Initialize a Vision Transformer model $M_{i}^{k}$ 9:     **for** *e* from 1 to *E* **do**10:        Train $M_{i}^{k}$ on $D_{train_{k}}$11:     **end for**12:   **end for**13:   Add $M_{i}^{k}$ to the ensemble list ensemble14:**end for**15:**Validation phase**:16:**for** *i* from 1 to *N* **do**17:   Initialize an empty list val_acc←[]18:   **for** *k* from 1 to *K* **do**19:     Compute validation accuracy $Acc_{i}^{k}$ of $M_{i}^{k}$ on $D_{val_{k}}$20:     Add $Acc_{i}^{k}$ to val_acc21:   **end for**22:   Compute the average validation accuracy $Acc_{i}$ for $M_{i}$ across all folds: $Acc_{i}=\frac{1}{K}\sum_{k=1}^{K}Acc_{i}^{k}$23:**end for**24:Select the model $M_{j}$ with the highest average validation accuracy $Acc_{j}$ as the final model25:**Return** $M_{j}$

### 4.3. Hyperparameter Settings

The hyperparameter settings used to generate Log Mel-spectrograms for each dataset are shown in Table 1. The value of each hyperparameter is determined empirically. The sampling rate is set to 22.5 kHz for UrbanSound8K and 44.1 kHz for ESC-10 and ESC-50. The frame size and window size are set to be the same for each dataset, with 2205 used for UrbanSound8K and 4410 used for ESC-10 and ESC-50. A larger window size is chosen to provide better frequency resolution, which is useful for accurately identifying the frequency content of signals. Additionally, longer windows result in fewer frames, reducing the computational load and memory requirements. For environmental sound classification tasks [46,47], longer windows are suitable for capturing distinctive spectral characteristics. The hop size is set to half of the frame size for each dataset, with 1103 used for UrbanSound8K and 2205 used for ESC-10 and ESC-50. The number of Mel bands used for all datasets is 128. All the resulting spectrograms have dimensions of 128×101, which are then resized into 3×224×224 before being fed into the member models. The input to each member model consists of the original Log Mel-spectrograms and their augmented counterparts generated using SpecAugment. The parameter for both time masking and frequency masking is set to 50.

The hyperparameter settings used to train the proposed EnViTSA model are shown in Table 2. The learning rate is set to 0.0001, the learning rate decay rate is 0.1, the weight decay rate is 0.001, the batch size is 32, and the dropout rate is 0.5. Training will automatically stop when the validation loss does not decrease for five consecutive epochs. The optimizer used is Adam. The weights of the MLP head are unfrozen in the Vision Transformer as the model has a large number of parameters to train.

### 4.4. Ablation Analysis

To perform ablation analysis, experiments without the use of augmented inputs and/or ensemble learning are also carried out for every dataset using the proposed EnViTSA.

Table 3 shows the comparison of validation accuracies obtained by the Vision Transformer for the UrbanSound8K, ESC-50, and ESC-10 datasets under different conditions. Ensemble learning has significantly improved the performance, while SpecAugment has slightly reduced the results. The combination of SpecAugment and ensemble learning has improved the results compared to when neither is applied, but the improvement is somewhat less significant than ensemble learning alone, possibly due to the negative effect brought about by SpecAugment or minor deviations between experiments, as the decrement is almost negligible.

### 4.5. Performance Evaluation and Analysis

Table 4 presents the average F1 scores for all classes in UrbanSound8K, as assessed across 10 iterations using an ensemble of five Vision Transformer models with SpecAugment. The F1 score is calculated for each class individually to evaluate the model’s performance in accurately identifying instances of each class. This approach offers a more comprehensive assessment of the model’s multiclass performance by considering its effectiveness for each specific class. The highest-performing class is gunshot, achieving an average F1 score of 92.92, demonstrating the model’s excellent ability to identify this sound class accurately. On the other end of the spectrum, the class air conditioner exhibits the lowest performance, with an average F1 score of 66.64, indicating challenges in correctly classifying this sound type. Street music and dog bark stand out as well-performing classes, boasting average F1 scores of 81.51 and 82.93, respectively. In contrast, jackhammer and engine idling are classified as poorly performing classes, with average F1 scores of 71.64 and 74.32, respectively. These F1 scores suggest that the model encounters more difficulty in accurately identifying sounds from these classes.

Table 5 provides an overview of the average F1 scores for all sound classes in the ESC-50 dataset across five iterations, employing an ensemble of five Vision Transformers with SpecAugment. These F1 scores offer insights into the model’s performance in accurately classifying each sound category. The highest-performing class, church bells, achieves an impressive average F1 score of 97.49, demonstrating the model’s proficiency in correctly identifying this particular sound class. In stark contrast, the class helicopter presents the lowest performance, with an average F1 score of 62.26, indicating significant challenges in classifying this specific sound category.

Notably, the model exhibits strong performance in several sound classes, including rooster, pouring water, thunderstorm, clock tick, glass breaking, flying insects, sea waves, clock alarm, siren, and car horn. These classes showcase average F1 scores that highlight the model’s ability to accurately recognize these sound types. Conversely, some sound classes demonstrate poor performance, such as wind, drinking/sipping, fireworks, breathing, door/wood creaks, engine, cat, water drops, and washing machine. These classes exhibit average F1 scores ranging from 71.26 to 78.37, indicating challenges in correct classification.

It is worth noting that the Vision Transformer model encounters difficulties with human and non-speech sounds, as evidenced by the classes from this category contributing to the 10 lowest average F1 scores. In contrast, none of these classes are included in the list of the 10 classes with the highest average F1 scores. In summary, these results suggest that the Vision Transformer model excels in classifying specific sound categories, particularly natural soundscapes and water sounds, while facing challenges with others.

Table 6 provides a comprehensive overview of the ensemble’s performance, featuring Vision Transformer models and SpecAugment on ESC-10—a subset of the ESC-50 dataset, consisting of 10 distinct sound classes. The performance evaluation is based on the calculation of average F1 scores, computed across five iterations.

Notably, the highest-performing class, clock tick, attains an exceptional average F1 score of 98.82. This result underscores the model’s remarkable ability to accurately identify every instance of this particular sound within the dataset. Conversely, the class dog demonstrates the lowest performance, achieving an average F1 score of 86.61. This outcome implies that the model encounters challenges in distinguishing this sound from other similar sounds present in the dataset.

In addition to clock tick, the model excels in correctly classifying crying baby and chainsaw, as evidenced by their high average F1 scores of 97.33. However, when dealing with helicopter, the model encounters difficulties, yielding a lower average F1 score of 88.11. These results collectively highlight the variability in performance exhibited by the Vision Transformer model and SpecAugment when applied to different sound classes within the ESC-10 dataset.

Table 7 presents a performance comparison of the proposed EnViTSA method with other existing methods on three different datasets: UrbanSound8K, ESC-50, and ESC-10. The proposed EnViTSA method outperforms all other methods on all three datasets, achieving a validation accuracy of 93.50% on UrbanSound8K, 85.85% on ESC-50, and 83.20% on ESC-10. This represents a significant improvement over the previous state-of-the-art methods, which achieved validation accuracies ranging from 56% to 92.5%.

In particular, EnViTSA outperforms EnvNet-v2, which was previously the best-performing method on ESC-50 and ESC-10, by a substantial margin. EnViTSA also achieves the highest accuracy on UrbanSound8K, surpassing ESResNet, which was previously the best-performing method on this dataset.

These results demonstrate the effectiveness of EnViTSA in environmental sound classification and suggest that the ensemble of Vision Transformer models with SpecAugment is a promising approach for improving classification performance. They offer valuable insights for researchers and practitioners working on environmental sound classification, providing a benchmark for evaluating different methods and highlighting the potential of EnViTSA for practical applications.

## 5. Conclusions

In this paper, we present an approach for classifying environmental sounds using an ensemble of Vision Transformer models with SpecAugment. We evaluate the proposed method on three publicly available datasets: UrbanSound8K, ESC-50, and ESC-10. The results demonstrate that our method outperforms state-of-the-art methods on all three datasets, achieving high accuracy and F1 scores. We also illustrate the effectiveness of SpecAugment in improving the classification performance of the model, especially on the ESC datasets. The ensemble learning approach with multiple members significantly contributes to the improved performance by enhancing the model’s ability to capture diverse and complex features from the dataset.

Our analysis of the F1 scores for each class in the datasets reveals that the Vision Transformer model performs well on natural soundscapes and water sounds but poorly on human and non-speech sounds. Additionally, ensemble models like EnViTSA can be computationally intensive, limiting their applicability in resource-constrained environments such as embedded systems or mobile devices where real-time processing is required. This insight can be valuable for future research in this area, highlighting the need for more specialized models for certain types of sounds.

In summary, our proposed approach provides a promising solution for environmental sound classification, with potential applications in noise pollution monitoring and wildlife conservation. The results of this paper showcase the capability of Vision Transformer models in this field and offer valuable insights for future research.

## Figures and Tables

**Table 1:** Hyperparameter settings during spectrogram generation.

	Sampling Rate (kHz)	Frame Size/Window Size	Hop Size	Mel Band
UrbanSound8K	22.5	2205	1103	128
ESC-10/ESC-50	44.1	4410	2205	

**Table 2:** Hyperparameter settings For training the proposed EnViTSA.

Hyperparameters	Settings
Learning Rate	0.0001
Learning Rate Decay Rate	0.1
Weight Decay Rate	0.001
Batch Size	32
Dropout Rate	0.5
Optimizer	Adam
Early Stopping Condition	5 Epochs, Validation Loss

**Table 3:** Ablation analysis for three benchmark datasets under different conditions.

	UrbanSound8K	ESC-50	ESC-10
**Conditions**	**Validation Accuracy (%)**		
No SpecAugment + No Ensemble	79.30	77.85	92.50
No SpecAugment + Ensemble	83.32	84.15	94.50
SpecAugment + No Ensemble	78.54	82.90	91.50
SpecAugment + Ensemble (Final Result)	83.20	85.85	93.50

**Table 4:** Average F1 score for classes in UrbanSound8K.

Class Label	Average
Air Conditioner	66.64
Car Horn	77.65
Children Playing	80.51
Dog Bark	82.93
Drilling	77.70
Engine Idling	74.32
Gun Shot	92.92
Jackhammer	71.64
Siren	77.03
Street Music	81.51
Overall Average	78.28

**Table 5:** Average F1 score for classes in ESC-50.

Class Label	Average
Dog	86.90
Rooster	93.17
Pig	81.63
Cow	86.77
Frog	84.93
Cat	77.83
Hen	82.95
Insects (flying)	90.57
Sheep	88.63
Crow	90.22
Rain	86.60
Sea waves	90.49
Crackling fire	84.46
Crickets	90.29
Chirping birds	87.33
Water drops	78.37
Wind	74.08
Pouring water	94.33
Toilet flush	95.29
Thunderstorm	93.16
Crying baby	89.26
Sneezing	87.16
Clapping	87.90
Breathing	71.26
Coughing	84.74
Footsteps	81.81
Laughing	82.01
Brushing teeth	91.31
Snoring	85.94
Drinking, sipping	74.14
Door knock	88.32
Mouse click	87.17
Keyboard typing	80.72
Door, wood creaks	75.46
Can opening	89.63
Washing machine	73.70
Vacuum cleaner	87.46
Clock alarm	97.14
Clock tick	91.06
Glass breaking	96.32
Helicopter	62.26
Chainsaw	88.60
Siren	95.14
Car horn	92.78
Engine	74.55
Train	84.72
Church bells	97.49
Airplane	80.62
Fireworks	72.02
Hand saw	82.56
Overall Average	85.43

**Table 6:** Average F1 score for classes in ESC-10.

Class Label	Average
Dog	86.61
Rooster	96.47
Rain	90.64
Sea Waves	93.46
Crackling Fire	95.27
Crying Baby	97.33
Sneezing	90.41
Clock Tick	98.82
Helicopter	88.11
Chainsaw	97.33
Overall Average	93.45

**Table 7:** Performance comparison of the proposed EnViTSA with other existing methods (validation accuracy %).

Model	UrbanSound8K	ESC-50	ESC-10
PiczakCNN [38]	80.50	64.50	73.70
EnvNet [18]	-	71.00	-
SB-CNN [17]	-	-	79.00
GoogLeNet [39]	86.00	73.00	-
AlexNet [39]	86.00	65.00	-
PiczakCNN+TEO-GTSC [40]	-	81.95	-
PiczakCNN+PEFBEs [41]	-	84.15	-
EnvNet-v2 [18]	91.30	84.70	78.30
Multiresolution 1D-CNN [42]	-	75.10	-
WSNet [43]	-	66.25	-
CNN [44]	77.00	49.00	-
TDSN [44]	56.00	-	-
MC-DCNNs + Multi-level Fusion [45]	87.60	73.10	75.10
ESResNet – from scratch [28]	92.50	81.15	81.31
**EnViTSA (Ours)**	**93.50**	**85.85**	**83.20**

## Data Availability

Not applicable.
